# *Raillietia auris* (Mesostigmata: Raillietiidae) in cattle in the state of Mato Grosso do Sul, Brazil

**DOI:** 10.1590/S1984-29612022032

**Published:** 2022-06-06

**Authors:** Fernanda Paula Veloso, Fernando de Souza Rodrigues, Renata Cunha Madureira, Eliane Mattos Piranda, Luiz Eduardo Roland Tavares, Fernando Paiva

**Affiliations:** 1 Programa de Pós-graduação em Ciências Veterinárias, Universidade Federal de Mato Grosso do Sul – UFMS, Campo Grande, MS, Brasil; 2 Departamento de Medicina Veterinária Preventiva, Universidade Estadual de Londrina – UEL, Londrina, PR, Brasil; 3 Ministério da Agricultura Pecuária e Abastecimento, Campo Grande, MS, Brasil.; 4 Instituto de Biociências, Universidade Federal de Mato Grosso do Sul – UFMS, Campo Grande, MS, Brasil

**Keywords:** Bovine otitis, Raillietia auris, prevalence, mites, Otite bovina, Raillietia auris, prevalência, ácaros

## Abstract

Parasitic otitis in cattle, caused by mites, has been reported from several continents. The present study aimed to determine the distribution, prevalence, intensity, mean intensity, and range of the agent in cattle in the state of Mato Grosso do Sul, Brazil. The samples were designed at random, with an acceptable margin of error of 3% and a confidence interval of 99%. A total of 449 animals were sampled immediately after slaughter from 34 different municipalities in the state using the technique of flushing both ear canals. Only *Raillietia auris* (Leidy, 1872) were found, with a prevalence of 98.6%, mean intensity of 53.78 mites/animal, and a range of 1-323. Impressively, the prevalence found was identical to another survey carried out 39 years ago in the same region. Details about the parasite intensity in different age categories of the animals are presented. The study demonstrates that the prevalence and intensity of infestation by *Raillietia auris* are high, and in older cattle are higher than young ones.

## Resumo

As otites parasitárias em bovinos, causadas por ácaros, são reportadas nos vários continentes. O presente trabalho teve como objetivos determinar a distribuição, prevalência, intensidade, intensidade média e amplitude parasitária do agente em bovinos, no estado de Mato Grosso do Sul, Brasil. As amostragens foram delineadas ao acaso, como margem de erro aceitável de 3% e intervalo de confiança de 99%. Foram amostrados 449 animais, imediatamente após o abate, e oriundos de 34 diferentes municípios do Estado, empregando-se a técnica de lavagem de ambos os canais auditivos. Apenas ácaros da espécie *Raillietia auris* (Leidy, 1872) foram encontrados, com uma prevalência de 98,6%, intensidade parasitária média de 53,78 ácaros/animal e amplitude de 1-323. Curiosamente, a prevalência encontrada é idêntica a outro levantamento realizado há 39 anos na mesma região. São apresentados detalhes sobre a intensidade parasitária em diferentes categorias de idade dos animais. O estudo demonstra que a prevalência e intensidade de infestação por *Raillietia auris* é alta, e bovinos mais velhos apresentam mais elevadas do que nos jovens.

## Introduction

Parasitic otitis in bovines can be caused by the ear mites *Raillietia auris* (Leidy, 1872) and *Raillietia flechtmanni* Faccini, Leite, Costa, 1992b and by rhabditiform nematodes, *Caenorhabditis bovis* (Kreis, 1964), *Metarhabditis blumi* (Sudhaus,1974), *Rhabditis freitasi* Martins Jr, 1985, and *Rhabditis costai* Martins Jr, 1985. The cattle ear mite *R. auris* has been described in America, Africa, Asia, Europe, and Australia (Menzies & Eads, 1957; Ladds et al., 1972; Domrow, 1980; Heffner & Heffner, 1983; Jubb et al., 1993; Krametter-Froetscher et al., 2006). In Brazil, the parasite has been reported in the states of Rio Grande do Sul, Rio de Janeiro, Mato Grosso do Sul, São Paulo, Minas Gerais, Paraná, Pará, Acre, and Amapá (Faccini et al., 1992a).

In the state of Mato Grosso do Sul, Larangeira et al. (1982) sampled 50 animals in 12 months, recovering 2593 mites and determining the prevalence of the infestation at 98%, with a mean intensity of 52.9, and a range of 1-120.

The life cycle in vitro is completed between 2 and 5 days (30 °C and 85-90% relative humidity), with the parasitic stages of egg, larva, protonymph, and deutonymph to adult (Fonseca & Faccini, 1985). The prevalence of infection increases with age, and *Bos indicus* breeds are more infested than others (Araújo et al., 1996). The mite is a parasite of the internal portion of the external ear canal and the external surface of the tympanic membrane. The clinical importance of the mite is not well established, with animals with the predominance of subclinical infection and with rare records with clinical severity, such as a case of perforating the tympanum, resulting in vestibular disease or suppurative otitis (Menzies & Eads, 1957; Ladds et al., 1972; Jubb et al., 1993; Krametter-Froetscher et al., 2006; Ferry et al., 2011).

The objective of the present study was to report the distribution, prevalence, and intensity of cattle infestation caused by *R. auris* in the state of Mato Grosso do Sul.

## Material & Methods

The study was conducted in a cattle slaughterhouse in Campo Grande, Mato Grosso do Sul, Brazil. The sample size was performed using the Epi Info™ CDC module Statcalc - Population Survey, considering the bovine population of 21 498 382 head for the state of Mato Grosso do Sul (IBGE, 2012); delineated in the form of simple random probability sampling, estimating an expected parasitological prevalence of 95%, with a confidence interval of 99% and 3% for an acceptable margin of error, which totaled a minimum of 350 samples, to meet the established requirements.

Data about sex was obtained with the archives of the official sanitation in the slaughterhouse. The diagnosis of the age of the animals was estimated by dentition according to Faísca et al. (2002).

The parasites were recovered by utilizing a modified technique first described by previous authors (Faccini et al., 1987; Araújo et al., 1996). During the slaughter process, the skulls, after the removal of skins and muscles and the separation of the lower jaw, were examined before being discarded to the rendering plant; a syringe was used to inject 50 mL of water flux into each ear canal. The mechanical action of the pressurized water would flush out the parasites present. The effluent was collected individually for each animal into a plastic container. In the laboratory, the sample was put into a conical sedimentation glass for concentration, and the parasites were observed, identified, and quantified under a stereomicroscope. Isolated mites were then preserved in Eppendorf tubes containing a 70 GL alcohol solution.

Some specimens were randomly selected among the different samples; a total of 40 individuals (25 female and 15 males) were individually transferred to standard 75 × 25 mm blank microscope slides with a round excavation in the center filled with a drop of Hoyer's solution, and a coverslip was placed over it after proper positioning. The specimens were kept at 45 °C for 24-26 hours and then examined for their taxonomic characters by light microscopy performed using a Leica DM5500 B™ microscope or a Leica M205 C™ stereomicroscope, both equipped with Leica cameras, models DFC 490 and 420™, respectively (Leica Microsystems™, Wetzlar and Mannheim, Germany), and images were registered by a system of Leica Application Suite image analysis – LAS™ 3.8 (Leica Microsystems™, Wetzlar and Mannheim, Germany). The taxonomic characters considered were those described previously by Trouessart (1902), Domrow (1980), Faccini et al. (1992b), and Ferry et al. (1999).

For scanning electron microscopy (SEM), the selected specimens were dehydrated in a progressive series of 70 to 99° GL ethyl alcohol, at 1-hour intervals between each dilution (70, 80, 90, and 99 GL). The specimens were then immersed in hexamethyldisilazane (Cat. Number 440191 Sigma-Aldrich ™) for 10 minutes followed by deposition onto Carbon Conductive Tabs, 12 mm OD, Pelco Tabs ™ (Ted Pella®, Inc., USA) adhered on Pelco® Q Pin Stub, 12.7 × 12.7 mm (Ted Pella®, Inc., USA). The images were documented using a Hitachi® Model TM3000 ™ Scanning Electron Microscope (Hitachi, Tokyo, Japan) in the analy mode.

Prevalence, intensity, mean intensity, and range were calculated as described by Bush et al. (1997). The data were not normally distributed (Kolmogorov Smirnov test, p < 0.05; data not shown). Therefore, the Kruskal-Wallis with Dunn post hoc test was applied for comparing the difference between the intensity of infestation by age categories of the cattle. Besides, Spearman's rank coefficient was used to calculate the correlation of parasite intensities between the age groups of the animals. Statistical analysis was performed using R (R Core Team R, 2020) language. Statistical differences among groups were considered significant at p < 0.05.

## Results

Four hundred and forty-nine animal samples (419 males and 30 females) were collected from cattle of 34 counties of the state of Mato Grosso do Sul. A total of 25,903 mites were recovered, predominantly composed of adult specimens and a few larvae; proto or deutonymph forms were not seen; eggs were observed in some samples. The prevalence of infested animals was 98.6%, and only six animals were not parasitized by *Raillietia* spp. The mean intensity was 53.78, and the range was 1-323, and a few animals presented purulent secretions, but still in the subclinical form of the infestation. Differences among counties and parasitic intensity were observed in this survey.

**Figure 1:**
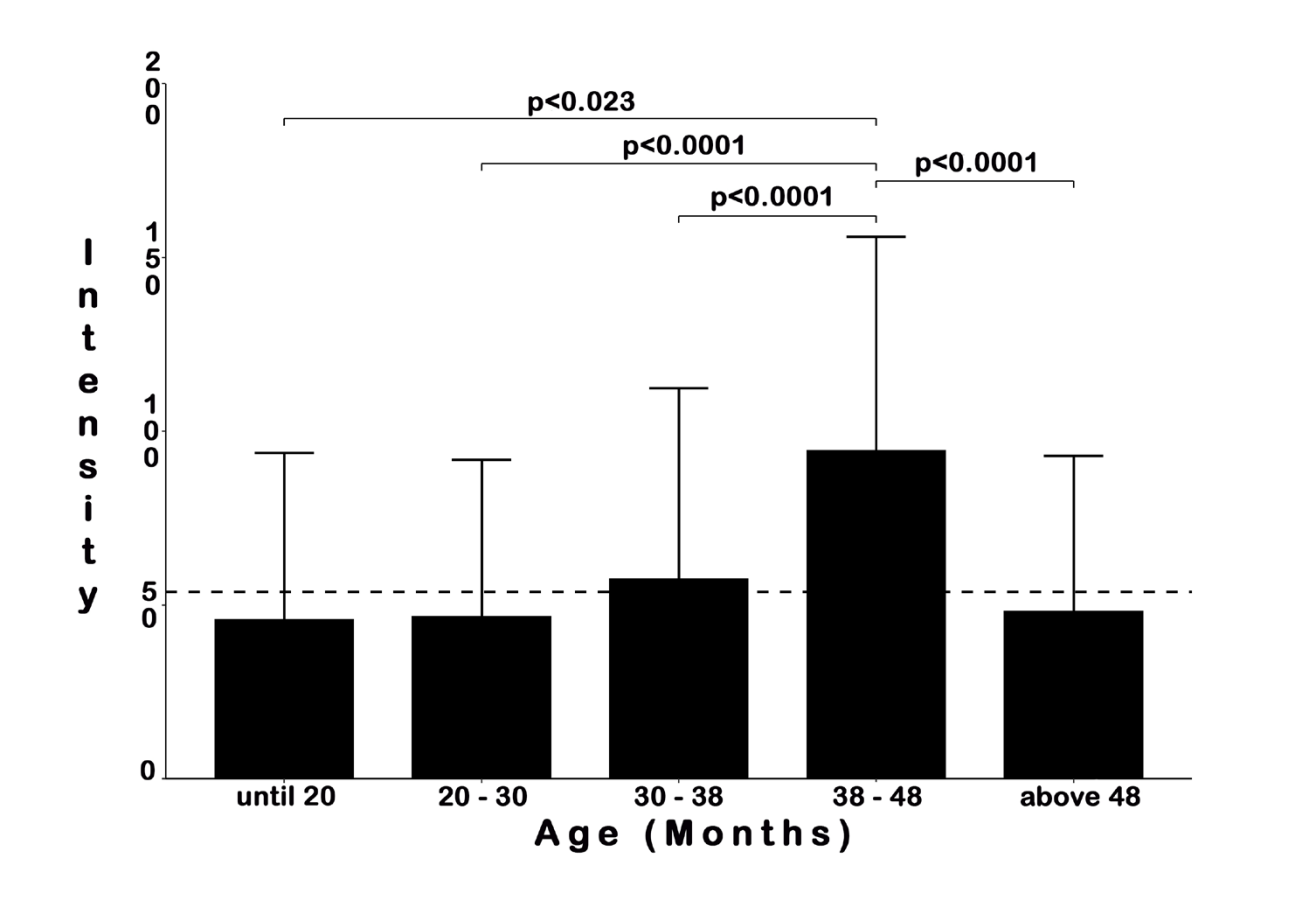
The intensity of *Raillietia auris* (Leidy, 1872) infestation amongst age groups of cattle in Mato Grosso do Sul, Brazil. The dashed line represents the mean intensity in the sampled animals. Statistical differences were considered significant at p < 0.05, analyzed by Kruskal-Wallis with Dunn *post hoc* test.

The age group with the higher intensity and mean intensity of infestation was among the animals 38-48 months old, with a significant difference for other ages groups (Figure 1). There was a weak significant positive correlation between parasite intensity and the age of the animals (rs = 0.1187, p = 0.0118), analyzed by Spearman´s rank coefficient. Twenty-nine (6.45%) animals presented purulent secretion simultaneously with parasitism by the mite. The nematodes were not found in the examined samples.

The specimens were identified as *Raillietia auris* (Leidy, 1872); and the following characters were observed: small idiosoma ovoid (1 mm), strongly domed, in the females, and males strongly humped. Both sexes with dorsal plates lozenge shaped. The gnathosoma, and the legs of a more or less dark reddish-brown, the rest of the body whitish. Dorsal shield with 12 pairs of setae. Chaetotaxy of tibia IV 2 1/1 3/1 1 in males and females.

Female with faintly colored sternal plate ending in an edge straight or slightly arched; faintly colored genital plate, narrower, with a fringed or strongly pleated anterior edge, with posterior edge round. Genital aperture transverse, opening between these two plates. Claw-shaped chelicerae slender, toothed.

**Figure 2:**
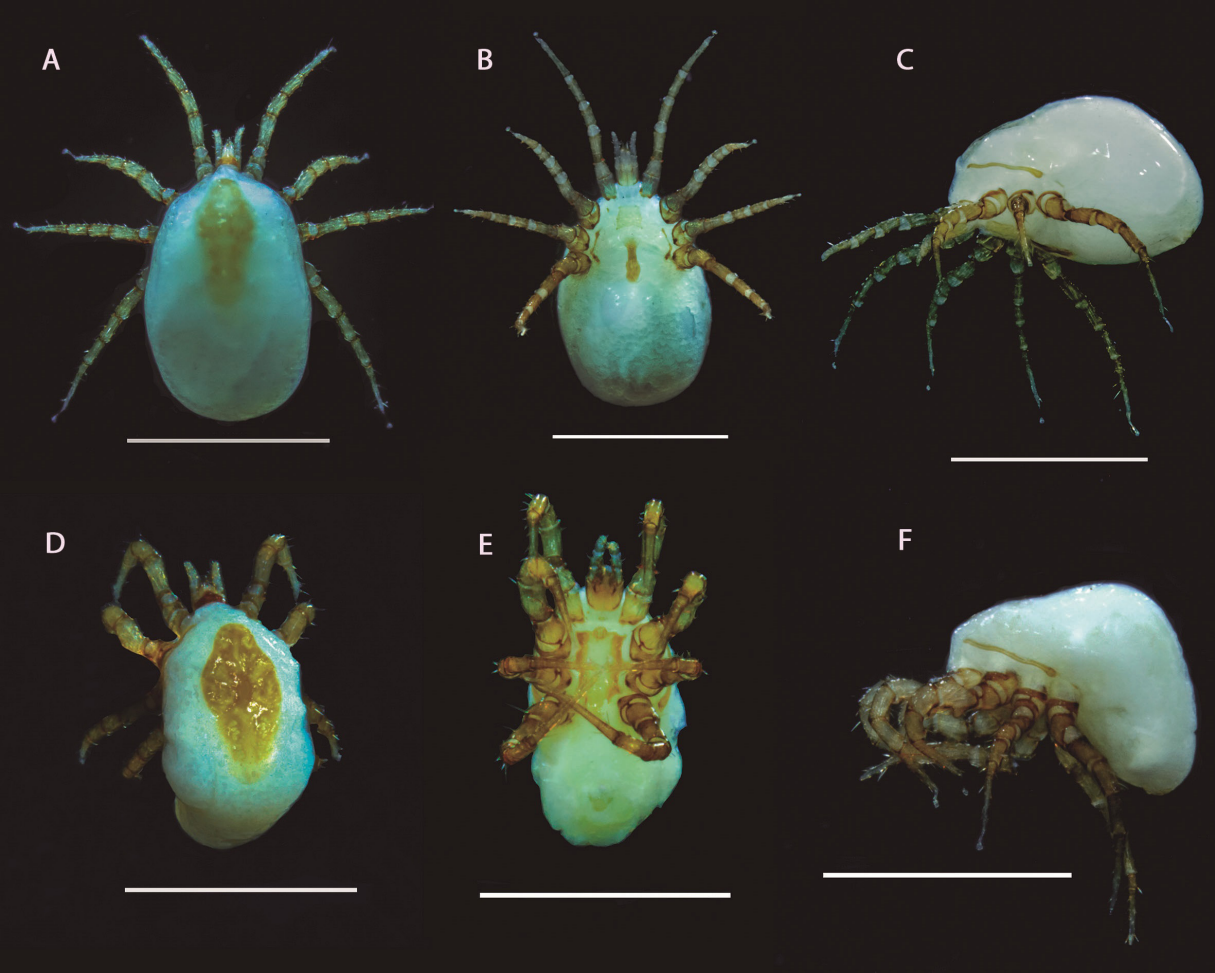
Stereomicroscopic images of specimens of *Raillietia auris* (Leidy, 1872) were collected from cattle and fixed in 70 GL ethyl alcohol. (A-C) female in dorsal, ventral and lateral view; (D-F) male in dorsal, ventral and lateral view. Scale bar= 1 mm.

**Figure 3:**
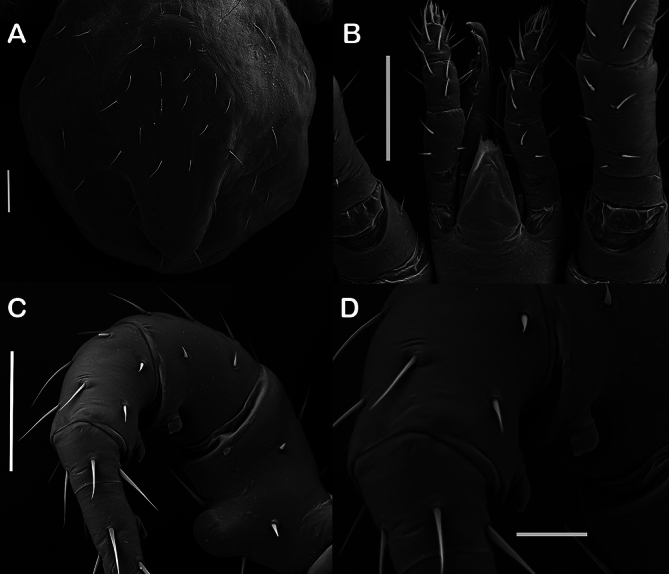
Scanning electron microscopy images of male specimens of *Raillietia auris* (Leidy, 1872) collected from cattle. (A) detail of the dorsal shield; (B) gnathosoma with detail of the chelicerae and palps; (C) ventral process on femur II and peg-like setae on genu, tibia and tarsus II, and (D) detail of the peg-like setae on genu and tibia II. Scale bar in A-C= 100 µm, and D= 20 µm.

Male showing legs of the second pair swollen, bearing on their inferior-internal face, one ventral peg-like seta on the tarsus, tibia and genu and a ventral process on the femur. Chelicerae are robust, compressed, strongly striated, and showing dark reddish-brown. Morphological images of the adult mites are shown in Figure 2 and details of the male are shown on Figure 3.

## Discussion

It is noteworthy that the observed prevalence rate of 98.6% of *R. auris* in cattle in the Mato Grosso do Sul state is exactly as reported thirty years ago (Larangeira et al., 1982). The current findings suggest that, regardless of the advancements made during the past three decades concerning the use of insecticides or mainly endectocides (macrocyclic lactones), the prevalence of the cattle ear mite remains virtually the same in the region.

Araújo et al. (1996) also found that the intensity of infestation increased with the age. Older cattle increase the accumulation of cerumen and other secretions in the ears (Duarte et al., 2001), which can contribute to maintaining a suitable environment for mites and nematodes.

The stages protonymph and deutonymph are free stages of the parasite, and transmission may occur by the teneral adult mites (Costa et al., 1992a), thus the environmental factors probably influence the transmission of the parasite.

Despite the high prevalence and considerable parasitic intensity recorded in the sampled regions, there are no published records on clinical cases of otitis - *stricto sensu* - caused by *R. auris* in cattle; occasionally there are comments from veterinarians about some events in which it is possible to infer that the mite is most likely involved due to the reported symptoms; however, these rare cases have not been verified and properly documented.

In general, infestations with *R. auris* are not commonly associated with clinical signs (Krametter-Froetscher et al., 2006) and have high specificity to the host (Costa et al., 1992b). The mite normally inhabits the external side of the tympanic membrane, with cattle supporting the infestation (Araújo et al., 1996) in such a way that the statement of Heffner & Heffner (1983) is presented as feasible: “the infestations of cattle with *R. auris* appeared to be the rule than the exception”.

However, it is consistent to assume that the ulceration and blockage of the ear canal by cerumen and pus must cause some discomfort to the animal (Heffner & Heffner, 1983). Ladds et al. (1972) reported the association of damage to the tympanic membrane with the presence of *R. auris*, and the authors stated that the mites may act with a primary cause for otitis media in their hosts. There is a record of signs of ear irritation, and even more serious signs of nervous symptoms such as circling, ataxia, unilateral facial paralysis, and lateral recumbency with loss of righting reflex have been reported (Jubb et al., 1993; Krametter-Froetscher et al., 2006).

In the few reports of treatment for *R. auris*, differences in efficacy were related; Duarte et al. (2001) did not find this species in healthy cattle raised under pasture and suggested that the intensive use of miticidal pour-on products (cypermethrin or ivermectin) for controlling ticks of the species *Rhipicephalus* (*Boophilus*) *microplus* (Canestrini, 1888) may have influenced the infestation. In Australia, treatments of calves with ivermectin (subcutaneously) and flumethrin (pour-on) were not effective against the mites in the ear canal 22 days from treatment; however, the use of flumethrin directly in the ear canal eliminates the mites (Jubb et al., 1993).

Cases of parasitic otitis in cattle caused by nematodes have some reports in Brazil (Martins, 1985; Leite et al., 1993; Duarte et al., 2001; Bossi et al., 2015); however, in this study, they were not found.

## Conclusion

The study demonstrates that the prevalence of and intensity of infestation by *R. auris* is high, 98.6%, and has remained at the same levels for over thirty years, and the mite is still present in different regions of the State of Mato Grosso do Sul. Older cattle have more parasites than young ones. It is still unknown which ecological factors can influence the maintenance of mite infestations in animals.
